# Glucose Biosensor Based on a Glassy Carbon Electrode Modified with Polythionine and Multiwalled Carbon Nanotubes

**DOI:** 10.1371/journal.pone.0095030

**Published:** 2014-05-09

**Authors:** Wenwei Tang, Lei Li, Lujun Wu, Jiemin Gong, Xinping Zeng

**Affiliations:** 1 Department of Chemistry, Tongji University, Shanghai, China; 2 School of Life Science and Technology, Tongji University, Shanghai, China; University of Akron, United States of America

## Abstract

A novel glucose biosensor was fabricated. The first layer of the biosensor was polythionine, which was formed by the electrochemical polymerisation of the thionine monomer on a glassy carbon electrode. The remaining layers were coated with chitosan-MWCNTs, GOx, and the chitosan-PTFE film in sequence. The MWCNTs embedded in FAD were like “conductive wires” connecting FAD with electrode, reduced the distance between them and were propitious to fast direct electron transfer. Combining with good electrical conductivity of PTH and MWCNTs, the current response was enlarged. The sensor was a parallel multi-component reaction system (PMRS) and excellent electrocatalytic performance for glucose could be obtained without a mediator. The glucose sensor had a working voltage of −0.42 V, an optimum working temperature of 25°C, an optimum working pH of 7.0, and the best percentage of polytetrafluoroethylene emulsion (PTFE) in the outer composite film was 2%. Under the optimised conditions, the biosensor displayed a high sensitivity of 2.80 µA mM^−1^ cm^−2^ and a low detection limit of 5 µM (S/N = 3), with a response time of less than 15 s and a linear range of 0.04 mM to 2.5 mM. Furthermore, the fabricated biosensor had a good selectivity, reproducibility, and long-term stability, indicating that the novel CTS+PTFE/GOx/MWCNTs/PTH composite is a promising material for immobilization of biomolecules and fabrication of third generation biosensors.

## Introduction

Glucose has always been an analyte that has received much attention because of its vital effect in clinical and environmental analysis. Since the first concept of an enzyme-based biosensor was proposed by Clark and Lyons [1], tremendous interest has been focused on the fabrication of glucose enzyme biosensors. The enzyme biosensor has experienced three stages in previous years. Due to its better selectivity and stability, the third-generation biosensor based on the direct electrochemistry of glucose oxidase has been widely developed [2]–[4].

However, for the flavin adenine dinucleotide (FAD) that is the active centre of glucose oxidase (GOx) and is deeply embedded in the molecular cavity [5], it is generally difficult to establish direct electron transfer (DET) between the active centre of GOx and conventional electrodes. To enhance this communication, nanomaterials have been extensively used in biosensors to achieve DET. Various nanomaterials composed of carbon [6], [7] metal oxides [8], [9] and conducting polymers [10], [11] have demonstrated the capabilities of DET. However, significant challenges remain in the fabrication of practicable biosensors that show superior bioelectrocatalysis, precise specificity, and high stability.

Multiwalled carbon nanotubes (MWCNTs) have unique electronic, mechanical, and thermal properties, which leads to their potential application in the fabrication of biosensors. Many recent reports have demonstrated that the integration of MWCNTs and conducting polymers [12], [13] or metal nanoparticles [14], [15] could greatly improve the direct electrochemistry of GOx.

Recently, conducting polymers have been extensively employed for the construction of electrochemical biosensors due to their conductive nature and superior stability [16]. Thionine (TH) is a phenothiazine dye (CH_3_COOC_12_H_10_N_3_S) with two amino groups symmetrically distributed on each side of its aromatic rings [17]. Under a suitable voltage, the aromatic rings of TH can be linked together via –NH– bridges to form polythionine (PTH) [18]. The PTH has good conductivity and exhibits a fast charge transfer capacity, showing great potential for various future applications [19], [20].

In this study, we investigated the step-wise immobilizations of PTH, MWCNTs, GOx, and chitosan-PTFE composite films onto a glassy carbon electrode to build a biosensor for measuring glucose. A mediator-free GOx-based glucose biosensor was constructed though a layer-by-layer self-assembly approach. Electrochemical impedance spectroscopy was used to monitor the biosensor modification process. DET between the active site of the GOx and the electrode was achieved, and its behaviour as a biosensor and its enzyme stability were investigated by electrochemical techniques.

## Experimental

### Reagents

Glucose oxidase (GOx, EC 1.1.3.4, 100 U/mg, from Aspergillus niger) and bovine serum albumin (BSA) were purchased from MP Biomedicals Co., Ltd. (Shanghai). The multiwalled carbon nanotubes (MWCNTs, >95% purity) were obtained from the Chengdu Organic Chemicals Co., Ltd. of the Chinese Academy of Science (Chengdu, China). Uric acid (UA) was purchased from Sigma. D-glucose, chitosan, glacial acetic acid, glutaraldehyde, vitamin C, L-cysteine (L-cys), and the polytetrafluoroethylene emulsion (60% PTFE) were of analytical grade and were purchased from Sinopharm Chemical Reagent Co., Ltd (SCRC, China).

### Apparatus and measurements

Amperometric experiments and cyclic voltammetric experiments were performed on an Autolab AUT72230 electrochemical work station (Metrohm China Ltd.). A conventional three-electrode system was applied: the modified or unmodified GCE electrode (3 mm diameter) acted as the working electrode, and a saturated calomel electrode (SCE) and a platinum wire served as the reference and counter electrodes, respectively. All of the electrochemical experiments were carried out at room temperature. Scanning electron microscopy (SEM) images were taken using a Tescan 5236 SEM (Tescan China Ltd.).

### Electrode modification

The glassy carbon electrode (GCE, Φ = 3 mm) was polished with 1.0 and 0.05 µm α-Al_2_O_3_ slurries successively and was then rinsed with water, acetone, absolute ethanol, and doubly-distilled water. According to the method described by Wang [21], the PTH/GCE was built and covered with a golden PTH film after 50 cycles of the electrodeposition of thionine (the potential range was between +0.1 and −0.55 V at 50 mV s^−1^).

Two mg MWCNTs was dispersed by ultrasonication in 1 mL 0.5% chitosan (CTS) solution. One mg GOx and 7.5 mg bovine serum albumin (BSA) were added to 100 µL 0.1 M PBS to prepare a 10 mg mL^−1^ GOx aqueous solution.

Preparation of the outer chitosan-PTFE composite film: the 60% (w/w) PTFE solution was dispersed by ultrasonication in a 0.5% chitosan solution, which was finally diluted to different ratios of 1%, 2%, 5%, and 10% (w/w) chitosan/PTFE suspension (CTS+PTFE).

**Figure 1:**
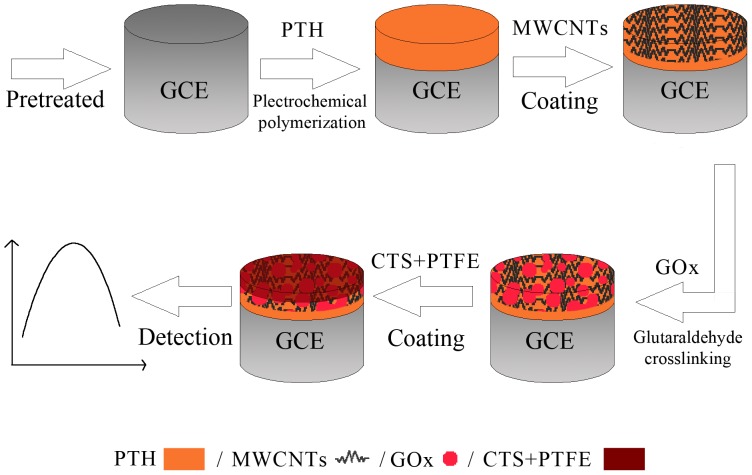
Schematic illustration of the stepwise fabrication process of CTS+PTFE/GOx/MWCNTs/PTH.

A CTS+PTFE/GOx/MWCNTs/PTH/GCE coating was prepared facilely by successively casting 6 µL 2 mg mL^−1^ MWCNTs, 6 µL 10 mg mL^−1^ GOx aqueous solution (using glutaraldehyde crosslinking as the fixation method), and 5 µL 2% (w/w) chitosan/PTFE suspension onto the pre-treated PTH/GCE. Each casting was performed after the previous cast had been air-dried. Finally, the modified GCE was immersed in PBS with a pH = 7.0 to remove the loosely absorbed GOx and was stored at 4°C under dry conditions. For comparison, MWCNTs/GCE,PTH/GCE,MWCNTs/PTH/GCE, and GOx/MWCNTs/GCE coatings were also prepared according to the same procedure. The assembly process of CTS+PTFE/GOx/MWCNTs/PTH is showed in Figure 1.

## Results and Discussion

### SEM characterisations of MWCNTs and electropolymerisation of thionine

#### SEM characterisation of the MWCNTs

**Figure 2:**
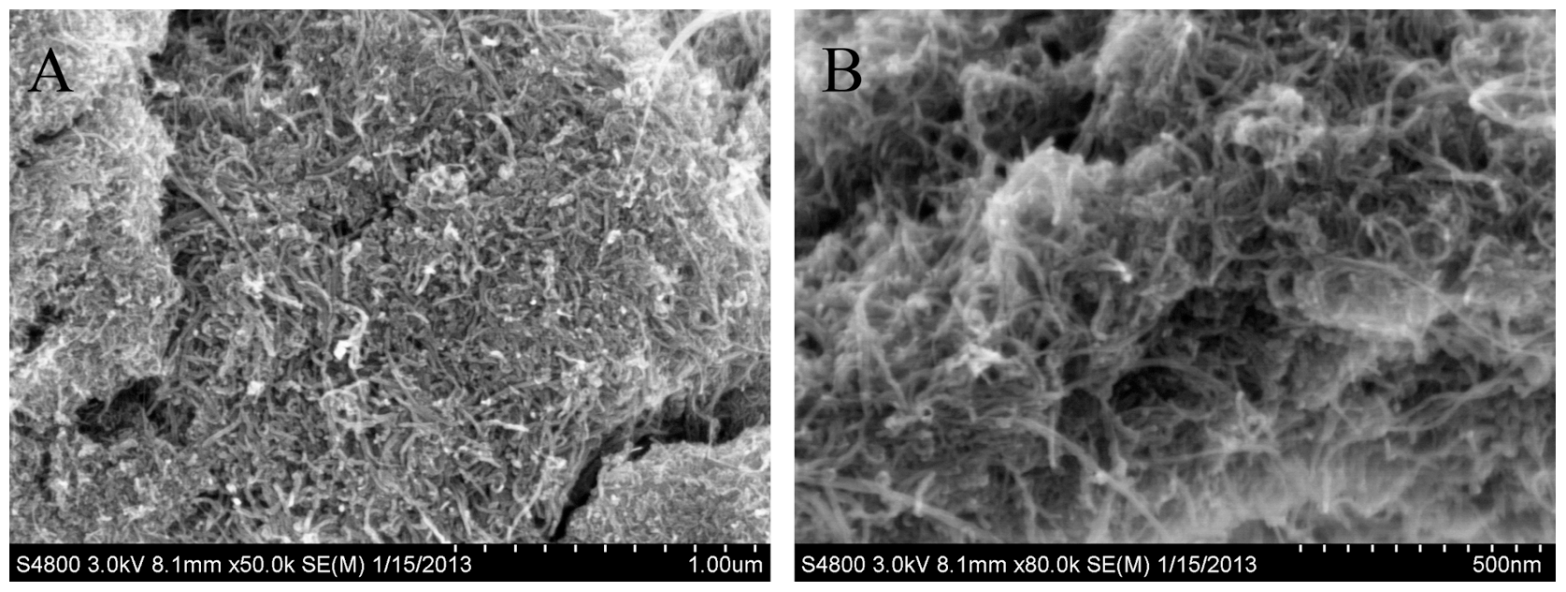
SEM images of the MWCNTs (A×50 k, B×80 k).

Figure 2 shows the SEM image of the MWCNTs deposited onto the glassy carbon surface. The disordered filamentous carbon nanotubes, with tube diameters of tens of nanometres, built a porous and three-dimensional structure, which can provide a conductive, porous, and biocompatible microenvironment for enzyme immobilization.

#### Electropolymerisation of thionine

**Figure 3:**
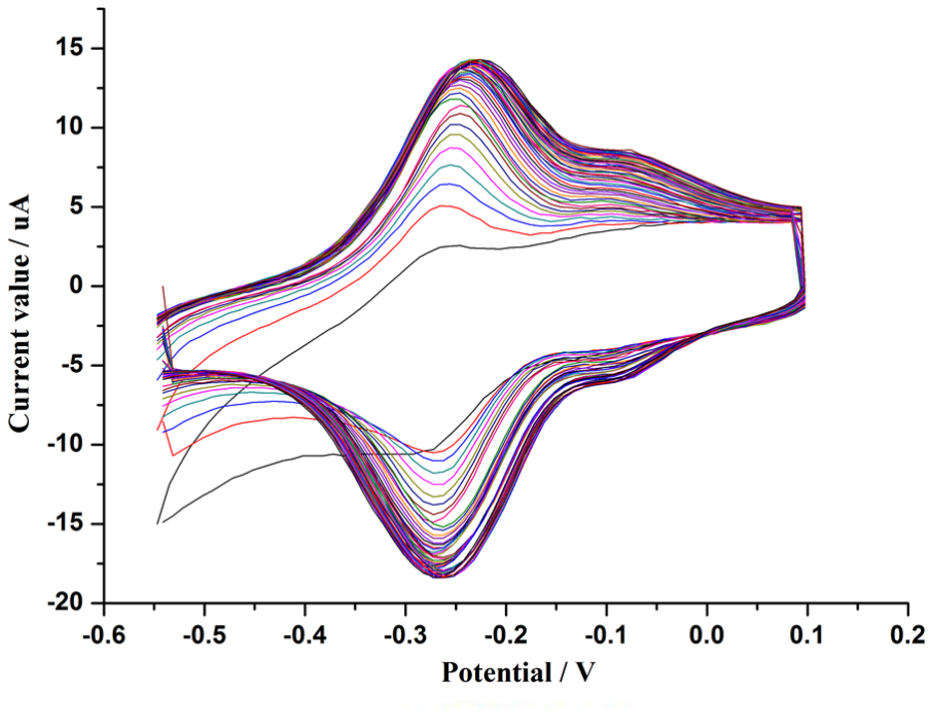
Cyclic voltammograms of the growth process of the PTH film with the sweeping potential between +0.1 and −0.55 V for 50 cycles (n = 1–50) in a pH = 6.5 PBS solution containing 0.5 mM thionine.

During the process of thionine electropolymerisation (Figure 3), the currents of a pair of reversible redox peaks increased gradually with increasing scan numbers, which can be attributed to the redox reaction of the PTH at the electrode. The anodic peak was located at −0.22 V and the cathodic peak was at −0.26 V after 50 scans. The results are very similar to Wang's study [21], and the electrode is also covered by a firm golden film after electropolymerisation. In addition, a derived redox peak was located at −0.07 V, which may be due to the differences in the raw materials.

### Electrochemical behaviour of CTS+PTFE/GOx/MWCNTs/PTH/GCE

EIS can give more detailed information on the impedance changes during the modification process. When presented in a Nyquist plot, the impedance spectrum includes a semi-circular portion corresponding to the electron-transfer-limited process and a linear part resulting from the diffusion process [22]. The diameter of the semicircle corresponds to the electron transfer resistance (RET) of the redox probe at the electrode interface. A smaller RET value implies that the probe has a higher interfacial electron transfer rate.

**Figure 4:**
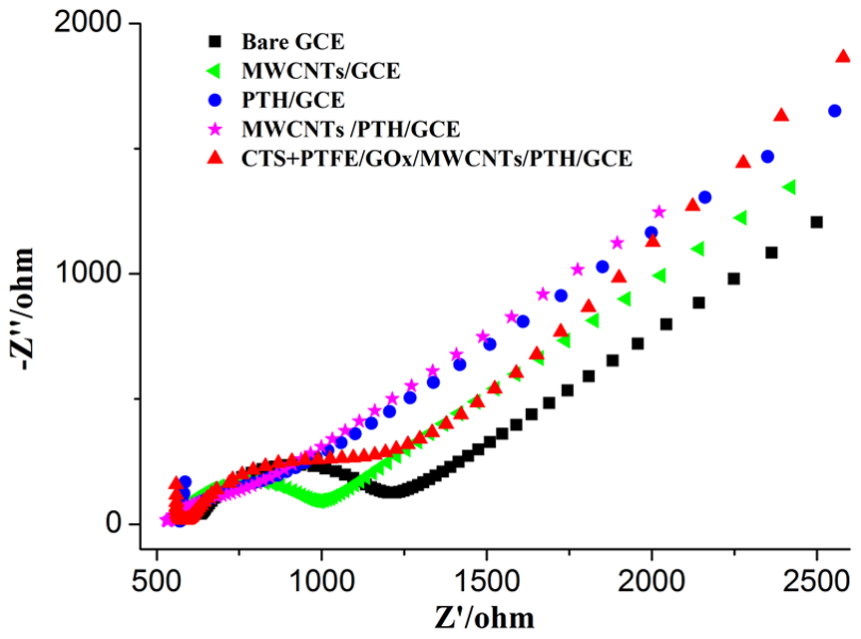
The electrochemical impedance spectra of bare GCE, MWCNTs/GCE, PTH/GCE, MWCNTs/PTH/GCE, and CTS+PTFE/GOx/MWCNTs/PTH/GCE. Supporting Electrolyte: a 1:1 solution of 0.1 M KCl containing 5.0 mM Fe (CN)_6_^3-/4-^.

Compared with bare GCE (Figure 4), the diameter of the semicircle greatly and successively decreased after the modification with MWCNTs/GCE, PTH/GCE, and MWCNTs/PTH/GCE, which indicated that both the MWCNTs and the PTH are good conductive materials, with the latter showing better conductive performance. The combination of MWCNTs and PTH on the GCE (MWCNTs/PTH/GCE) can greatly reduce the electrode resistance and promote a higher electron transfer rate between the redox probe and the electrode surface. However, an increase in the electron transfer resistance was observed after CTS+PTFE/GOx was immobilised on the probe, suggesting a decrease in the conductivity of CTS+PTFE/GOx/MWCNTs/PTH/GCE due to the immobilization of non-conductive PTFE and biomacromolecules. The results of the impedance change during the electrode modification can provide evidence for the successful immobilization of MWCNTs, PTH, GOx, and other materials on the electrodes.

**Figure 5:**
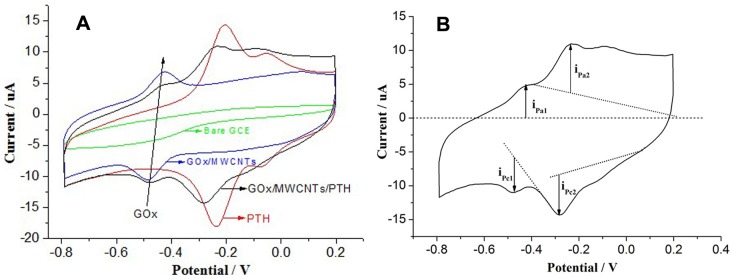
Cyclic voltammograms (CVs) of bare GCE, PTH/GCE, GOx/MWCNTs/GCE, and CTS+PTFE/GOx/MWCNTs/PTH/GCE in 0.1 M N_2_-saturated PBS (pH = 7.0) at a scan rate of 50 mV s^−1^, from −0.8 V to 0.2 V (A); Analysis of the CVs of CTS+PTFE/GOx/MWCNTs/PTH/GCE in Fig. 5 A (B).

Figure 5 (A) shows that for the GCE, there was no obvious redox peaks; two pairs of distinct redox peaks (−0.2 V and −0.07 V, respectively) were observed for PTH/GCE, which were confirmed as the characteristic peaks of PTH according to its electropolymerisation. For GOx/MWCNTs/GCE, there was a pair of standard redox peaks, whose reduction and oxidation peak potentials were −0.443 V and −0.471 V, respectively. These peak potentials were very close to the standard electrode potential of GOx, which is derived from the FAD/FADH_2_ GOx redox cofactor [23], [24]. The results also proved that the MWCNTs can directly promote electron transfer between GOx and the electrode. At −0.45 V, −0.27 V, and −0.13 V, three pairs of well-defined and nearly symmetric redox peaks were observed in GOx/MWCNTs/PTH. This result indicated that both GOx and PTH maintained good redox activities and that the modification layers provided a proper “micro room” for GOx. Both of the redox peak potentials were negatively shifted, which may be due to the parallel multi-component reaction system (PMRS). On the one hand, the redox species on the biosensor kept their separate redox characteristics; on the other hand, they were interdependent in the modification layers as a whole. Due to the high-impedance of GOx and PTFE, the peak current value of PMRS declined compared with PTH/GCE. The results are consistent with the previous electrochemical impedance spectral study.

From Figure 5 (B), it can be observed that the ratio of the GOx oxidation and reduction peaks is close to 1 (i_pa1_/i_pc1_≈1) and ΔEp_1_≈30 mV; however, that ratio for PTH is slightly greater than 1 (i_pa2_/i_pc2_>1) and ΔEp_2_≈20 mV. These results further demonstrated the good reversibility of PMRS.

**Figure 6:**
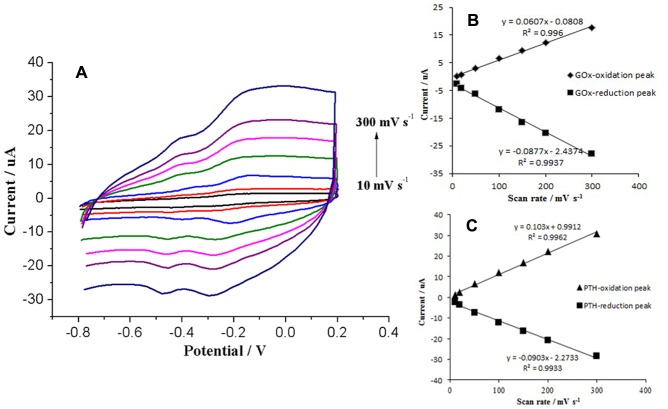
CVs of the CTS+PTFE/GOx/MWCNTs/PTH/GCE electrode in 0.1 M air-saturated PBS, at scan rates of 10, 20, 50, 100, 150, 200, and 300 mV s^−1^ (A); The value of the enzyme peak current as it changes with the scanning speed (B); The value of the PTH peak current as it changes with the scanning speed (C).

Both the anodic and cathodic peak currents of GOx and PTH increased linearly with an increase in the scan rate from 10 to 300 mV s^−1^ (Figure 6 (A), (B), and (C)), which is characteristic of a surface-controlled quasi-reversible electrochemical process [25]. These results suggested that GOx and PTH were immobilised as adsorbed reactants on the electrode surface.

### Biocatalytic activity of GOx on the biosensor

**Figure 7:**
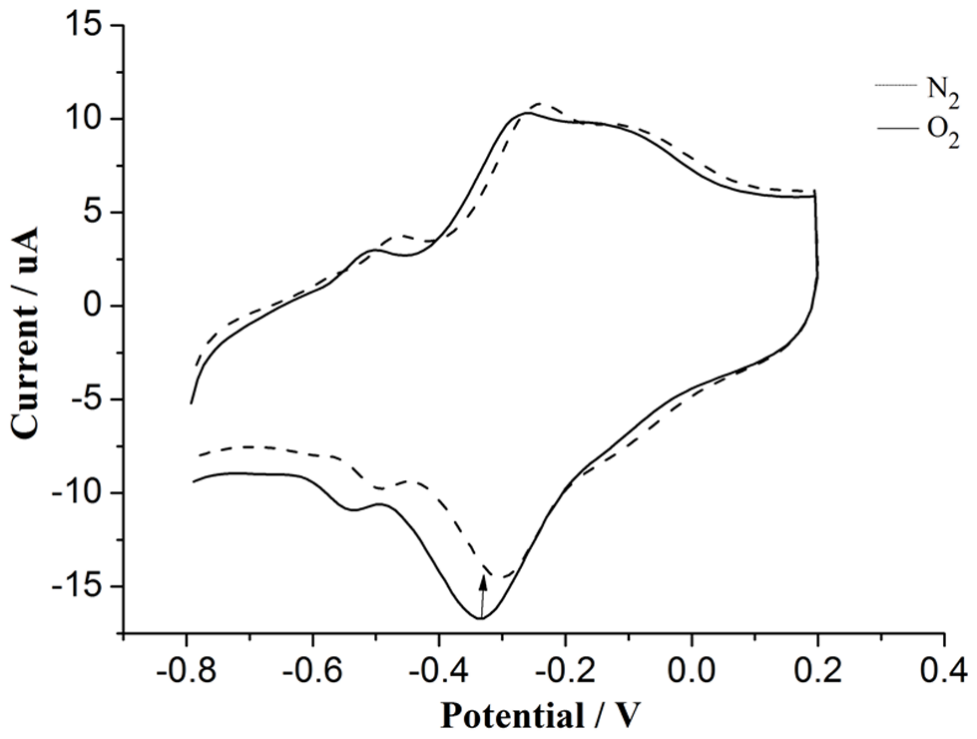
Cyclic voltammograms of the electrode modified with CTS+PTFE/GOx/MWCNTs/PTH/GCE in N_2_-saturated and air-saturated 0.1 M PBS with a pH = 7.0.

The electrocatalytic activity of GOx for oxygen was convincing proof of the biocatalytic activity of the GOx immobilised on the electrode surface [26]. From Figure 7, three pairs of redox peaks were observed in both N_2_-saturated and air-saturated PBS solutions. Compared to the peak currents of both GOx and PTH in the corresponding N_2_-saturated solution, an increase in the cathodic peak current and decrease in anodic peak current were observed in the air-saturated solution. This response can be explained as follows:
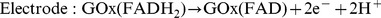
(1)


In N_2_-saturated PBS, DET occurred between the active site of the enzyme and the stable bioelectrode. 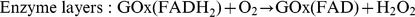
(2)


When in an air-saturated solution, the electrochemically formed GOx (FADH_2_) catalysed the reduction of oxygen dissolved in the solution. The relative consumption of GOx (FADH_2_) and generation of GOx (FAD) directly led to the changes in the peak currents of GOx. Because GOx and PTH were both in the PMRS of the electrode, the peak currents of PTH had the same tendency to change, which can be used as an indicator of the change in GOx in turn.

### Glucose Sensing Performance of CTS+PTFE/GOx/MWCNTs/PTH/GCE

**Figure 8:**
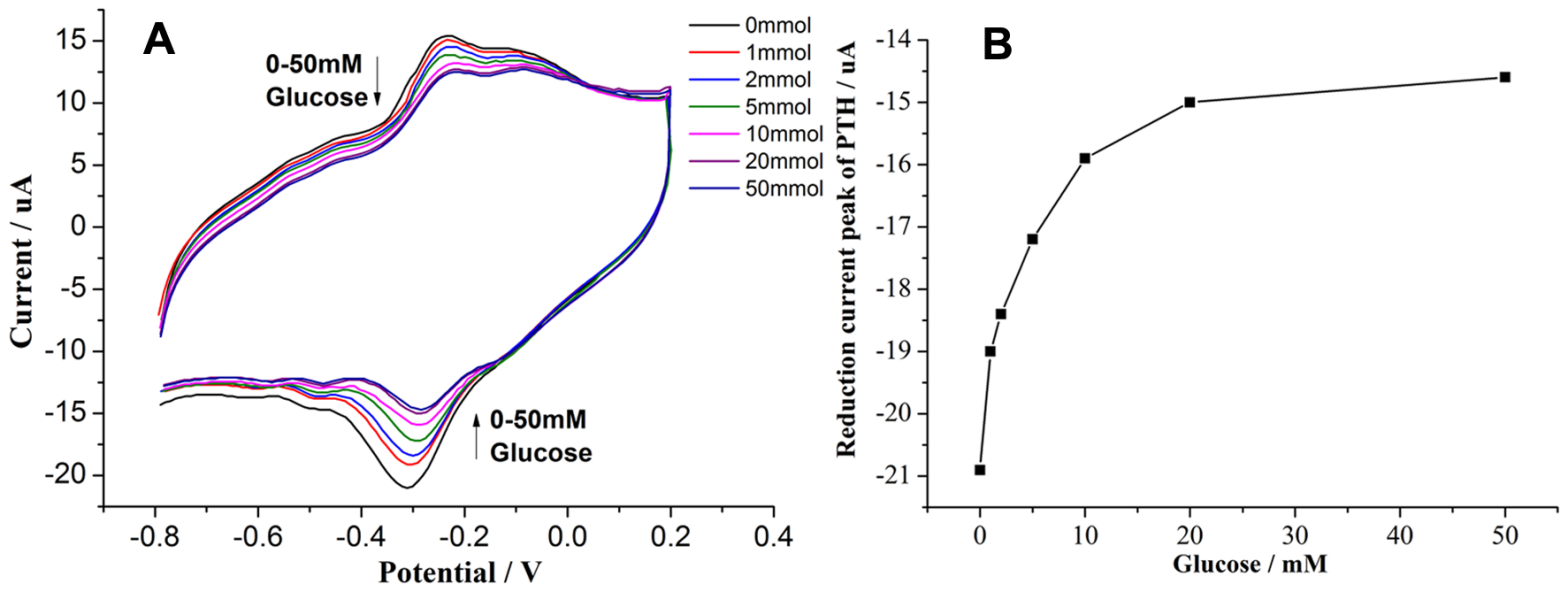
CVs of the CTS+PTFE/GOx/MWCNTs/PTH-modified biosensor in 0.1 M N_2_-saturated PBS solution (pH = 7.0) with different glucose concentrations (0, 1, 2, 5, 10, 20, and 50 mM glucose) (A); The plot of the reduction current peak of PTH vs. the glucose concentrations (B).

In Figure 8 (A), three pairs of redox peaks (GOx located at −0.45 V and PTH located at −0.3 V) were observed in the PBS without glucose. With increases in the glucose concentration, both the anodic and cathodic peak currents decreased, which can be explained by the following reactions: 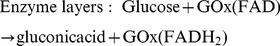
(3)

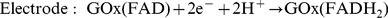
(4)


The equation indicates that GOx (FAD) is directly involved in the glucose oxidation reaction. As the glucose concentration increases, GOx (FAD) is competitively employed by glucose and increasingly converted into GOx (FADH_2_), leading to a continuous decrease in the cathodic and corresponding anodic peak currents of both GOx and PTH.

Figure 8 (B) depicts the plot of the cathodic peak currents of PTH vs. the glucose concentrations. The cathodic current decreased obviously with an increasing glucose concentration up to 10 mM. When the glucose concentration continued to increase, the current decreased slowly and finally flattened out. This result arose from GOx consumption and is similar to the research results of Si Peng [14], which suggested that the GOx on the surface of such an electrode is able to simultaneously demonstrate DET with the electrode and to retain its catalytic activity towards glucose. The sensing mechanism in our system is different from the first and second generation glucose sensors.

**Figure 9:**
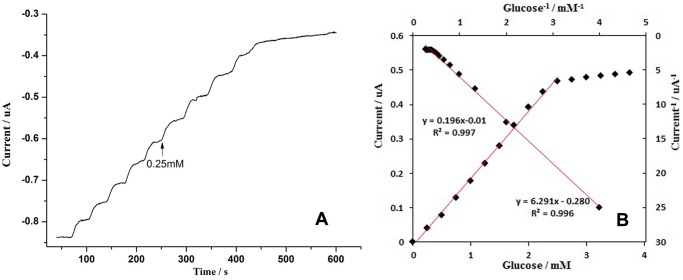
Amperometric response of the modified electrode in a stirred 0.1(pH = 7.0) for successive additions of 0.25 mM glucose at −0.42 V (A); the calibration curve (current vs. glucose concentration) and the Lineweaver Burk plot (current^−1^ vs. concentration^−1^) [27] obtained from the amperometric response in Fig. 9A (B).

Figure 9 (A) shows that each injection of 0.25 mM glucose resulted in a decrease of 50 nA in the cathodic current for the first 10 additions, which gave rise to a sensitivity of 2.80 µA mM ^−1^ cm^−2^. In addition, the current decreased steeply and then reached 95% of the steady-state current in less than 15 s. The current verses time plot showed a well-defined behaviour typical of an enzymatic reaction with a linear range up to 2.5 mM, and a current plateau was observed when the glucose concentration was higher than 2.5 mM, which suggests typical enzymatic reaction kinetics [28], [29].

The calibration curve (current vs. glucose concentration) and the Lineweaver Burk plot (current^−1^ vs. concentration^−1^) were obtained from the amperometric response (Figure 9 (B)). The linear regression equation of I (µA) = 0.196 (µA mM^−1^) C (mM)–0.01 (µA) (R = 0.997) was derived from the calibration curve and revealed that the glucose sensor has a detection limit as low as 5.0 µM (S/N = 3).

The apparent Michaelis-Menten constant *K_M_* was calculated using the electrochemical version of the Lineweaver Burk equation: 
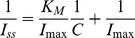
(5)where *I_ss_* is the steady-state current, *I_max_* is the saturation current, and C is the glucose concentration. *K_M_* was determined to be 3.1 mM from the intercept and slope of the Lineweaver Burk plot (Figure 9 (B)), which is much lower than the values of 8.5 mM for GOx immobilised on literature angle nanotubes [30], 13.9 mM for GOx immobilised on silica/multi-walled carbon nanotube/polyacrylonitrile nanocomposite layer redox polymers [31], and 14.6 mM for GOx immobilised on gold nanoparticles [32]. The smaller *K_M_* indicates that this biosensor has a superior enzymatic activity and a higher affinity for the glucose substrate [33].

### Optimization conditions for the glucose detection system

**Figure 10:**
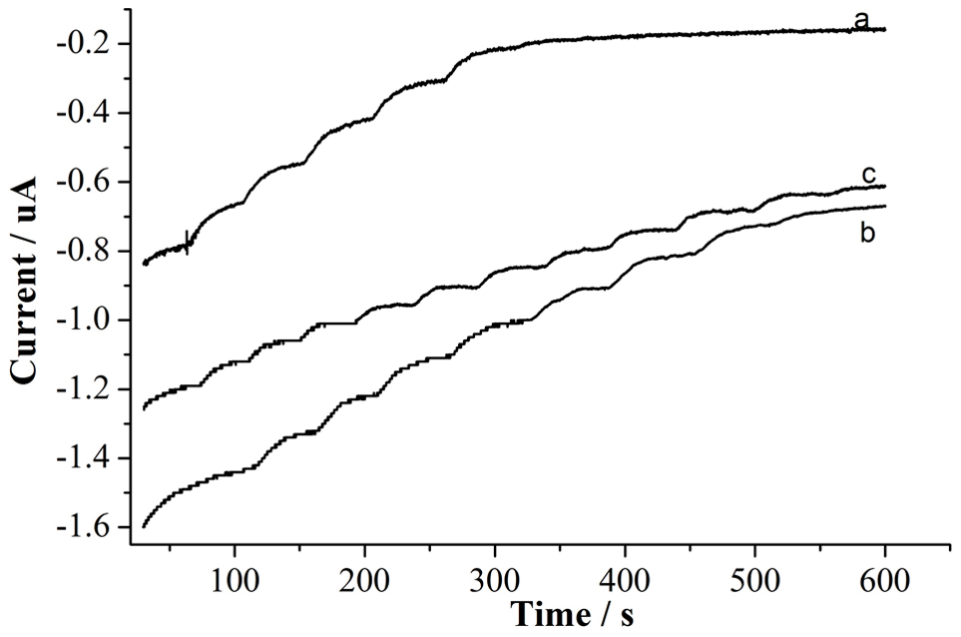
Amperometric response of CTS/GOx/MWCNTs/GCE (A), CTS/GOx/MWCNTs/PTH/GCE (B), and CTS+PTFE/GOx/MWCNTs/PTH/GCE (C) in a 0.1 M PBS (pH = 7.0) solution being stirred for successive additions of 0.25 mM glucose.

As shown in Figure 10, the sequence of the absolute differences between the starting and ending current values was ∣Ic_start_−Ic_end_∣<∣Ia_start_−Ia_end_∣<∣Ib_start_−Ib_end_∣, which was attributed to the combination of differently modified materials. For the glucose detection range, the size order was Step C>Step B>Step A, which showed that the biosensor using the CTS+PTFE/GOx/MWCNTs/PTH/GCE composite had a better amperometric response. The reason was that the CTS+PTFE film had good permeability and could make glucose molecules diffuse into the electrode surface smoothly and orderly.

We also did a series of research experiments to optimise the working conditions of the glucose sensor (the related figures and data are not shown). The biosensor has an optimum working voltage of −0.42 V, an optimum working temperature of 25°C, and an optimum working pH of 7.0. In addition, the best percentage of PTFE in the outer composite film was 2%.

### Selectivity, reproducibility, and stability of the glucose biosensor

**Figure 11:**
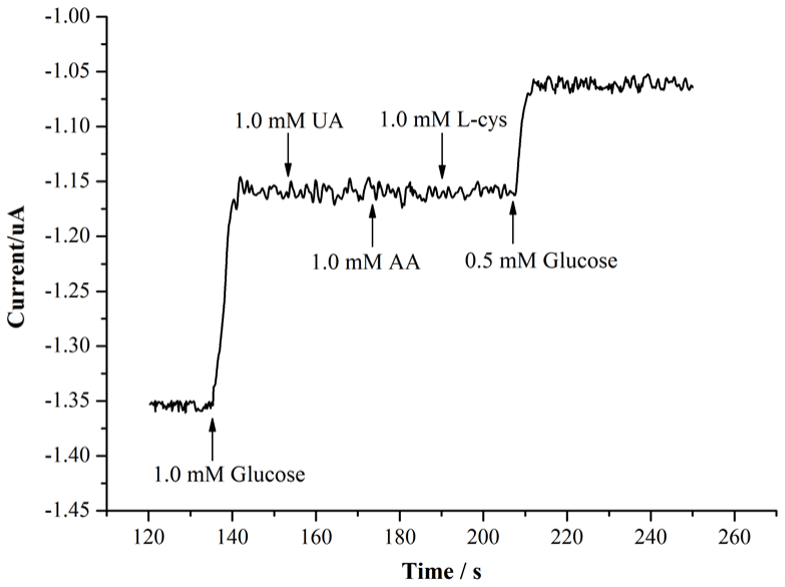
Amperogram showing the effects of interfering compounds (1.0 mM UA, 1.0 mM AA, and 1.0 mM L-cysteine) on the detection of glucose in 0.1 M PBS solution (pH = 7.0).

To evaluate the selectivity of the biosensor, many interfering biomolecules, including uric acid (UA), ascorbic acid (AA), and L-cysteine (L-cys), that normally co-exist with glucose in real samples (human blood) were investigated (Figure 11). The current generated from the interfering species, such as 1.0 mM of UA, AA, and L-cysteine, was negligible, indicating that the biosensor had a strong anti-interference ability. This strong anti-interference ability can mainly be attributed to the low working voltage and reduction method we adopted in this study, which can effectively avoid the oxidation of the interfering substances at high voltages.

**Figure 12:**
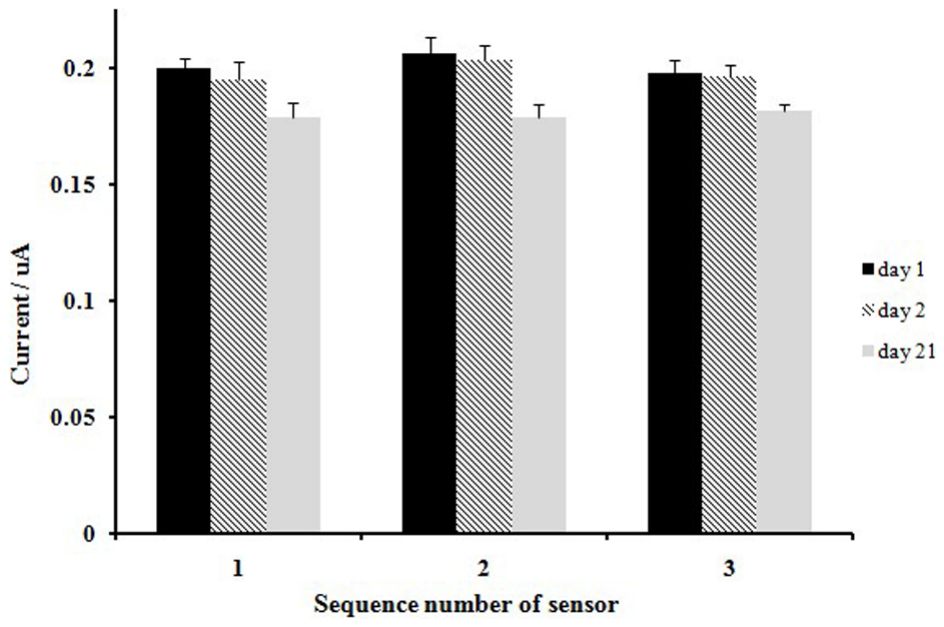
The stability of three modified biosensors over a storage period of 3 weeks in 0.1(pH = 7.0) with 1 mM glucose.

We put three sensors in PBS solution, which had a glucose concentration of 1.0 mM, to perform current tests. Then, the sensors were saved in the refrigerator at 4°C to perform stability tests. As Figure 12 shows, the current response had no obvious changes at the beginning after 2 days. After 3 weeks, the current responses decreased to 89.6%, 86.7%, and 91.7% of the initial response. In Figure 12, the relative standard deviation (RSD) was small and the reproducibility was good. The biosensor displayed an acceptable stability, which can be attributed to the following two properties: the immobilization of the MWCNTs and the thionine electropolymerisation on the electrode surface were very stable, which can provide a friendly environment for GOx and maintain its bioactivity; and the CTS+PTFE composite can effectively prevent enzyme leakage and prolong the life of the biosensor.

### The mechanism of DET of the sensor

**Figure 13:**
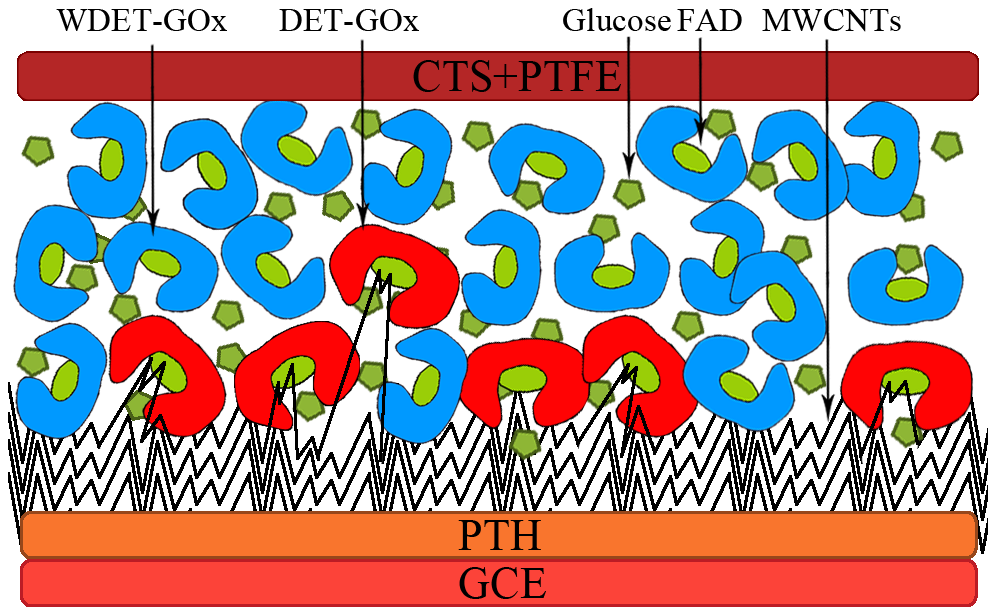
Distribution of DET-GOx and WDET-GOx in CTS+PTFE/GOx/MWCNTs/PTH.

In Figure 13, the active center of GOx (FAD) is buried deeply, only a few GOx can occur DET (DET-GOx), and the other part is just loaded up without DET-GOx (WDET-GOx). Both of them have biological catalysis activity, and can catch glucose and oxide them.

The current response of CTS+PTFE/GOx/MWCNTs/PTH is from the competitive reaction of glucose. Glucose competitively spreads to DET-GOx and reacts with it, thereby the DET-GOx is reduced, and thus the response current is decreased.

DET-GOx influences detection performance. The MWCNTs embedded in FAD are like “conductive wires” connecting FAD with electrode, reduce the distance between them and are propitious to DET. Combining with good electrical conductivity of PTH and MWCNTs, the current response is enlarged. According to response current, glucose concentration can be detected.

## Conclusions

In this work, we successfully introduced a promising glucose biosensor based on the immobilization of GOx onto a MWCNTs/PTH nanocomposite film. The evaluation of the CTS+PTFE/GOx/MWCNTs/PTH/GCE demonstrated that DET of GOx was achieved on this nanocomposite film. Cyclic voltammetry showed three pairs of well-defined redox peaks corresponded to DET of GOx (FAD/FADH_2_) and PTH. The parallel multi-component reaction system (PMRS) of the electrode played an important role in facilitating the electron transfer between GOx and the electrode surface. To the best of our knowledge, this is the first report about the direct electrochemistry of GOx based on PMRS. Furthermore, the potential use of the resulting electrode as a third generation glucose biosensor was confirmed by the amperometric response for successive additions of glucose. The biosensor showed a high sensitivity, fast amperometric response and low detection limit. Possible interfering species in blood, such as uric acid, ascorbic acid, and L-cysteine, did not influence the glucose determination. In addition, the CTS+PTFE film had good permeability and could greatly improve glucose detection range and stability. The MWCNTs embedded in FAD were like “conductive wires” connecting FAD with electrode, reduced the distance between them and were propitious to DET. Combining with good electrical conductivity of PTH and MWCNTs, the current response was enlarged. These results demonstrate that the CTS+PTFE/GOx/MWCNTs/PTH is an attractive material for the fabrication of efficient amperometric biosensors. The method presented can be used for the immobilization and evaluation of DET of other enzymes or proteins.

## References

[1] Clark LC, Lyons C (2006) Electrode systems for continuous monitoring in cardiovascular surgery. Ann N Y Acad Sci 102: 29–45. 10.1111/j.1749-6632.1962.tb13623.x 14021529

[2] Wu X, Zhao F, Varcoe JR, Thumser AE, Avignone-Rossa C, et al (2009) Direct electron transfer of glucose oxidase immobilized in an ionic liquid reconstituted cellulose-carbon nanotube matrix. Bioelectrochemistry 77: 64–68. 19535301 10.1016/j.bioelechem.2009.05.008

[3] Guo CX, Li CM (2010) Direct Electron Transfer of Glucose Oxidase and Biosensing of Glucose on Hollow Sphere-Nanostructured Conducting Polymer/Metal Oxide Composite. Phys Chem Chem Phys 12: 12153–12159. 20714592 10.1039/c0cp00378f

[4] Wang YL, Liu L, Li MG, Gao F (2011) Multifunctional carbon nanotubes for direct electrochemistry of glucose oxidase and glucose bioassay. Biosens Bioelectron 30: 107–111. 21959226 10.1016/j.bios.2011.08.038

[5] Wilson R, Turner APF (1992) Glucose-oxidase—an ideal enzyme. Biosens Bioelectron 7: 165–185.

[6] Wu S, Ju HX, Liu Y (2007) Conductive Mesocellular Silica Carbon Nanocomposite Foams for Immobilization, Direct Electrochemistry, and Biosensing of Proteins. Adv Funct Mater 17: 585–592.

[7] Gooding JJ, Wibowo R, Liu JQ, Yang WR, Losic D, et al (2003) Protein Electrochemistry Using Aligned Carbon Nanotube Arrays. J Am Chem Soc 125: 9006–9007. 15369344 10.1021/ja035722f

[8] Peng HP, Liang RP, Qiu JD (2011) Facile Synthesis of Fe_3_O_4_@Al_2_O_3_ Core-Shell Nanoparticles and their application to the highly specific capture of heme proteins for Direct Electrochemistry. Biosens Bioelectron 26: 3005–3011. 21185712 10.1016/j.bios.2010.12.003

[9] Zhu ZH, Qu LN, Niu QJ, Zeng Y, Sun W, et al (2011) Urchinlike MnO_2_ Nanoparticles for the Direct Electrochemistry of Hemoglobin with Carbon Ionic Liquid Electrode. Biosens Bioelectron 26: 2119–2124. 20926275 10.1016/j.bios.2010.09.017

[10] Wang ZY, Liu SN, Wu P, Cai CX (2009) Detection of Glucose Based on Direct Electron Transfer Reaction of Glucose Oxidase Immobilized on Highly Ordered Polyaniline Nanotubes. Anal Chem 81: 1638–1645. 19170516 10.1021/ac802421h

[11] Guo CX, Li CM (2010) Direct Electron Transfer of Glucose Oxidase and Biosensing of Glucose on Hollow Sphere Nanostructured Conducting Polymer/Metal Oxide Composite. Phys Chem Chem Phys 12: 12153–12159. 20714592 10.1039/c0cp00378f

[12] Wu XE, Zhao F, Varcoe JR, Thumser AE, Avignone-Rossa C, et al (2009) Direct electron transfer of glucose oxidase immobilized in an ionic liquid. Bioelectrochemistry 77: 64–68. 19535301 10.1016/j.bioelechem.2009.05.008

[13] Deng CY, Chen JH, Nie Z, Si SH (2010) A sensitive and stable biosensor based on the direct electrochemistry of glucose oxidase assembled layer-by-layer at the multiwall carbon nanotube-modified electrode. Biosensors and Bioelectronics 26: 213–219. 20620040 10.1016/j.bios.2010.06.013

[14] Si P, Ding SJ, Yuan J, Lou XW, Kim DH (2011) Hierarchically structured one-dimensional TiO_2_ for protein immobilization,direct electrochemistry, and mediator-free glucose sensing. ACS Nano 5: 7617–7626. 21866956 10.1021/nn202714c

[15] Wang Y, Yuan R, Chaia YQ, Li WJ, Zhuo Y, et al (2011) Direct electron transfer: Electrochemical glucose biosensor based on hollow Pt nanosphere functionalized multiwall carbon nanotubes. Journal of Molecular Catalysis B: Enzymatic 71: 146–151.

[16] Shirsat MD, Too CO, Wallace GG (2008) Amperometric glucose biosensor on layer by layer assembled carbon nanotube and polypyrrole multilayer film. Electroanal 20: 150–156.

[17] Li QW, Zhang J, Yan H, He MS, Liu ZF (2004) Thionine-mediated chemistry of carbon nanotubes. Carbon 42: 287–291.

[18] Bauldreay JM, Archer MD (1983) Dye-modified electrodes for photogalvanic cells. Electrochim Acta 28: 1515–1522.

[19] Zhang KY, Zhang L, Xu JK, Wang C, Geng T, et al (2010) A sensitive amperometric hydrogen peroxide sensor based on thionin/EDTA/carbon nanotubes—chitosan composite film modified electrode. Microchim Acta 171: 139–144.

[20] Liu Y, Zhang HL, Lai GS, Yu AM, Huang YM, et al (2010) Amperometric NADH biosensor based on magnetic chitosan microspheres/poly(thionine) modified glassy carbon electrode. Electroanal 22: 1725–1732.

[21] Wang QX, Zhang HL, Wu YW, Yu AM (2012) Amperometric hydrogen peroxide biosensor based on a glassy carbon electrode modified with polythionine and gold nanoparticles. Microchim Acta 176: 279–285.

[22] Bard AJ, Faulkner LR (2001) Electrochemical methods: Fundamentals and applications, 2nd edn. John Wiley&Sons, New York.

[23] Liu G, Paddon MN, Row JJ (2007) A molecular wire modified glassy carbon electrode for achieving direct electron transfer to native glucose oxidase. Electrochem. Commun 9: 2218–2223.

[24] You CP, Li X, Zhang S, Kong JL, Zhao DY, et al (2009) Electrochemistry and biosensing of glucose oxidase immobilized on Pt-dispersed mesoporous carbon. Microchim Acta 167: 109–116.

[25] Gao RF, Zheng JB (2009) Amine-terminated ionic liquid functionalized carbon nanotube-gold nanoparticles for investigating the direct electron transfer of glucose oxidase. Electrochem Commun 11: 608–611.

[26] Zhang HF, Meng ZC, Wang Q, Zheng JB (2011) A novel glucose biosensor based on direct electrochemistry of glucose oxidase incorporated in biomediated gold nanoparticles-carbon nanotubes composite film. Sensor Actuat B-Chem 158: 23–27.

[27] Murthy ASN, Sharma J (1998) Glucose oxidase bound to self-assembled monolayers of bis(4-pyridyl) disulfide at a gold electrode: Amperometric determination of glucose. Anal Chim Acta 363: 215–220.

[28] Kamin RA, Wilson GS (1980) Rotating ring-disk enzyme electrode for biocatalysis kinetic studies and characterization of the immobilized enzyme layer. Anal Chem 52: 1198–1205.

[29] Manesha KM, Kim HT, Santhosh P, Gopalan AI, Lee KP (2008) A novel glucose biosensor based on immobilization of glucose oxidase into multiwall carbon nanotubes-polyelectrolyte-loaded electrospun nanofibrous membrane. Biosens Bioelectron 23: 771–779. 17905578 10.1016/j.bios.2007.08.016

[30] Liu XQ, Shi LH, Niu WX, Li HJ, Xu GB (2008) Amperometric Glucose Biosensor Based on Single-Walled Carbon Nanohorns. Biosens Bioelectron 23: 1887–1890. 18387291 10.1016/j.bios.2008.02.016

[31] Nenkova R, Ivanova D, Vladimirova J, Godjevargova T (2010) New amperometric glucose biosensor based on cross-linking of glucose oxidase on silica gel/multiwalled carbon nanotubes/polyacrylonitrile nanocomposite film. Sensor Actuat B-Chem 148: 59–65.

[32] German N, Ramanaviciene A, Voronovic J, Ramanavicius A (2010) Glucose biosensor based on graphite electrodes modified with glucose oxidase and colloidal gold nanoparticles. Microchim Acta 168: 221–229.

[33] Guo CX, Li CM (2010) Direct Electron Transfer of Glucose Oxidase and Biosensing of Glucose on Hollow Sphere-Nanostructured Conducting Polymer/Metal Oxide Composite. Phys Chem Chem Phys 12: 12153–12159. 20714592 10.1039/c0cp00378f

