# Correction: Glucose Biosensor Based on a Glassy Carbon Electrode Modified with Polythionine and Multiwalled Carbon Nanotubes

**DOI:** 10.1371/journal.pone.0119971

**Published:** 2015-03-25

**Authors:** The PLOS ONE Staff

There is an error in the final sentence of the Apparatus and Measurements subsection of the Experimental section. The correct sentence is: Scanning electron microscopy (SEM) images were taken using a Hitachi S-4800 scanning electron microscope.
